# A randomized controlled trial of an intervention for infants’ behavioral sleep problems

**DOI:** 10.1186/s12887-015-0492-7

**Published:** 2015-11-13

**Authors:** Wendy A. Hall, Eileen Hutton, Rollin F. Brant, Jean Paul Collet, Kathy Gregg, Roy Saunders, Osman Ipsiroglu, Amiram Gafni, Kathy Triolet, Lillian Tse, Radhika Bhagat, Joanne Wooldridge

**Affiliations:** University of British Columbia School of Nursing, T 201, 2211 Wesbrook Mall, Vancouver, BC V6T 2B5 Canada; Midwifery Education Program, McMaster University, Michael G DeGroote Centre for Learning & Discovery, Room 2210, 1200 Main Street West, Hamilton, ON L8N 3Z5 Canada; Department of Statistics, University of British Columbia/Child and Family Research Institute, ESB 3146, 2207 Main Mall, Vancouver, BC V6T 1Z4 Canada; Child and Family Research Institute, University of British Columbia, Clinical Support Building, Room V3-320, 948 West 28th Avenue, Vancouver, BC V6H 3N1 Canada; University of British Columbia School of Nursing, Vancouver, BC Canada; University of British Columbia Faculty of Medicine, 2329 West Mall, Vancouver, BC V6T 1Z4 Canada; British Columbia Children’s Hospital, Division of Developmental Pediatrics, 3644 Slocan Street, Vancouver, BC V5M 3E8 Canada; Department of Clinical Epidemiology and Biostatistics, Faculty of Health Sciences, McMaster University, 1280 Main Street West, CRL-208, Hamilton, ON L8S 4K1 Canada; Pacific Spirit Community Health Centre, Vancouver Coastal Health, 2110 43rd Avenue West, Vancouver, BC V6M 2E1 Canada; South Community Health Centre, Vancouver Coastal Health, 6405 Knight Street, Vancouver, BC V5P 2V9 Canada; South Community Health Centre, Vancouver Community, Vancouver Coastal Health, 6405 Knight Street, Vancouver, BC V5P 2V9 Canada; Early Childhood Development at Vancouver Coastal Health, Vancouver Coastal Health, 11th floor, 601 West Broadway, Vancouver, BC V5Z 4C2 Canada

**Keywords:** Randomized controlled trial, Multi-component intervention, Behavioral sleep problems, Infants, Actigraphy, Diaries, Parents

## Abstract

**Background:**

Infant behavioral sleep problems are common, with potential negative consequences. We conducted a randomized controlled trial to assess effects of a sleep intervention comprising a two-hour group teaching session and four support calls over 2 weeks. Our primary outcomes were reduced numbers of nightly wakes or parent report of sleep problem severity. Secondary outcomes included improvement in parental depression, fatigue, sleep, and parent cognitions about infant sleep.

**Methods:**

Two hundred thirty five families of six-to-eight month-old infants were randomly allocated to intervention (*n* = 117) or to control teaching sessions (*n* = 118) where parents received instruction on infant safety. Outcome measures were observed at baseline and at 6 weeks post intervention. Nightly observation was based on actigraphy and sleep diaries over six days. Secondary outcomes were derived from the Multidimensional Assessment of Fatigue Scale, Center for Epidemiologic Studies Depression Measure, Pittsburgh Sleep Quality Index, and Maternal (parental) Cognitions about Infant Sleep Questionnaire.

**Results:**

One hundred eight intervention and 107 control families provided six-week follow-up information with complete actigraphy data for 96 in each group: 96.9 % of intervention and 97.9 % of control infants had an average of 2 or more nightly wakes, a risk difference of −0.2 % (95 % CI: −1.32, 0.91). 4 % of intervention and 14 % of control infants had parent-assessed severe sleep problems: relative risk 0.3, a risk difference of −10 % (CI: 0.11, 0.84-16.8 to −2.2). Relative to controls, intervention parents reported improved baseline-adjusted parental depression (CI: −3.7 to −0.4), fatigue (CI: −5.74 to −1.68), sleep quality (CI: −1.5 to −0.2), and sleep cognitions: doubts (CI: −2.0 to −0.6), feeding (CI: − 2.1 to - 0.7), anger (CI: − 1.8 to - 0.4) and setting limits (CI: −3.5 to −1.5).

**Conclusions:**

The intervention improved caregivers' assessments of infant sleep problem severity and parental depression, fatigue, sleep, and sleep cognitions compared with controls.

**Trial registration:**

ISRCTN42169337, NCT00877162

## Background

Behavioral sleep problems (BSP) affect 20 to 30 % of infants and often persist from infancy to later childhood [1, 2]. Predominant problems identified by parents are infants’ difficulties in falling asleep and staying asleep [2], which create fragmented sleep and/or short sleep duration. Adequate sleep duration is associated with increased infant adaptability and rhythmicity [3], while night waking with crying has been associated with greater stress reactivity, for example, during inoculations [4]. Young children’s BSPs have been linked to hyperactivity, and cognitive, emotional, and behavioral difficulties, after controlling for maternal depressive symptoms [5–9]. Infants’ BSPs have been associated with maternal depressive symptoms, serious psychological distress, poor general health, and feelings of incompetence [10–12]. Fathers, although rarely studied, have reported poorer general health and more psychological stress when their infants have BSPs [11].

A review of over 50 published treatment studies for children’s BSPs indicated behavioral interventions were efficacious, with over 80 % of children treated demonstrating clinically significant improvement that was maintained for 3 to 6 months [13]. Reviewers recommended future studies test group interventions and incorporate actigraphy as an objective sleep measure, measures of parental depression, and combined objective and subjective (parental diary) sleep measures [13]. Our before-after design pilot study of a cognitive-behavioral group intervention (CBGI) for seven groups of five parents (*n* = 35) with 6-to-12-month-olds experiencing BSPs demonstrated significantly reduced numbers of night wakes and longer night sleep time, by actigraphy, post intervention [14], as well as significantly improved parental mood, sleep quality, fatigue, and cognitions about infant sleep [15]. The CBGI involved a two-hour teaching session and two weeks of telephone support. Parents in that study reported that participating in a group teaching session with other parents whose children had sleep problems ‘normalized’ what they were experiencing and helped them regard their children’s sleep problems as common and amenable to change. Being part of the group and receiving telephone support calls increased their confidence to manage infant sleep [16].

In Canada, public health units are located in communities and deliver population-based programs and services that protect and promote Canadian’s health and focus on primary prevention [17]. Services vary by province and health authorities. In Vancouver, British Columbia, public health nurses have brief contact with healthy infants at birth, with no further formal contact unless families attend immunization clinics or postnatal drop-ins. Postnatal drop-ins offer families information about children’s nutrition and safety, adjusting to parenthood, and sleep [18]. Sleep sessions have provided general information about infant sleep and overviews of potential approaches to managing infant sleep. Our randomized controlled trial aimed to determine the efficacy of a cognitive-behavioral group intervention, offered through public health nurses. Our hypotheses were: fewer intervention group infants would have ≥2 night wakes or fewer intervention group parents would identify infants as having severe sleep problems at 6 weeks post-teaching session than control group parents. Secondary outcomes, at 6 weeks post-teaching session, were that parental mood, sleep quality, fatigue, and cognitions about infant sleep would improve significantly more in the intervention group and intervention group infants would have significantly longer longest sleep periods (actigraphy) and significantly fewer night wakes (actigraphy and sleep diary) than control group infants.

## Methods

### Ethics statement

The University of British Columbia (H09-00757) and Vancouver Coastal Health (#CS09-076) Research Ethics Boards approved the trial. Informed consent was provided by all participants, after obtaining written and oral information about the study. The consent form included the statement that the parents consented to their infant’s participation in the study. A data and safety monitoring board met annually to monitor adverse events. None occurred. No changes were made to the trial after commencement. The trial was registered on the following sites: ISRCTN, 42169337, url: http://www.isrctn.com/ NCT00877162, url: https://clinicaltrials.gov/.

### Participants and procedures

Posters, media announcements, online classifieds (Kijiji and Craigslist), and Facebook were used to disseminate information to families in the Greater Vancouver area about the trial. Posters were placed in coffee shops, libraries, community centres, and public health units. Interested families contacted the study coordinator to ask questions and be screened for inclusion. Families were told that we were testing an intervention to see whether it would improve infants’ BSP and that they would be assigned to receive the intervention or a control session on infant safety.

Eligible families were biological or adoptive, able to read and speak English, had access to a telephone, and constituted a single or two-parent family. Both parents in a two-parent family had to commit to participation in the study. Eligible infants had no identified health problems, were between corrected ages of five-point-five and eight months at recruitment, and were either sex. The study coordinator used a list of questions to determine whether infants met the inclusion criterion of demonstrating moderate BSP, as defined by the American Association of Sleep Medicine clinical Classification of Sleep Onset Association disorder (waking two or more times per night at least five nights per week) [19]. Parents who reported depression or sleep problem diagnoses or treatment, worked permanent night shifts, or lived outside the study catchment area (Greater Vancouver) were excluded. We excluded infants with organic causes of sleep disruption, developmental disability, or chronic neurological or respiratory conditions.

At baseline, research assistants delivered questionnaires (demographic, depression, sleep quality, sleep cognitions, and fatigue measures), actigraphs, and study-designed sleep diaries to families. Research assistants (blinded to group assignment) demonstrated how to apply actigraphs and complete the record of actigraph application and removal and sleep diaries. They also provided an information sheet that explained when to begin and end collecting actigraphic and diary data simultaneously. The research assistants left the questionnaires with the parents for self-administration. Baseline actigraphic and diary measurements occurred over 6 days. The first families were enrolled in September 2009; the final outcome assessment was completed in September 2011.

**Fig. 1:**
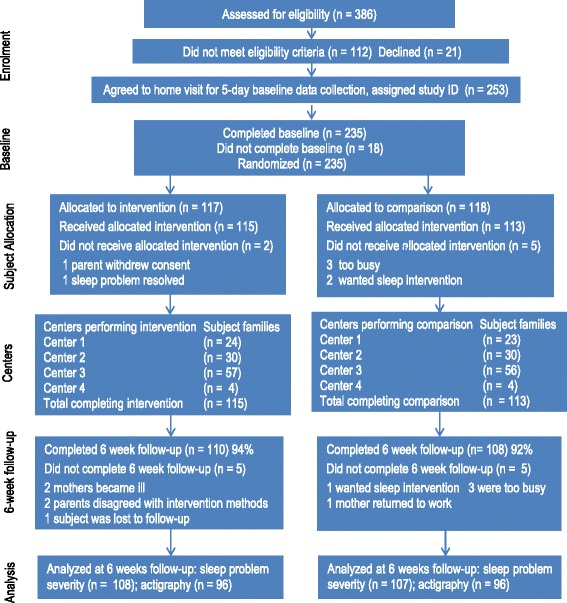
Participant Flowchart (CONSORT)

After the study coordinator enrolled families and research assistants obtained consent and baseline measurements, families were randomly allocated in sequential blocks of 10 to intervention (sleep) and control groups (safety) at each site (Fig. 1). The sequential blocks were to ensure that families were equally distributed by group designation and geographic area (4 health unit sites). A data manager used a computerized secure software platform to randomly assign families to groups.

Families attended a teaching session at one of 4 health units and received follow-up phone calls from the nurse who taught the session. Families from outside the health unit geographical boundaries attended a teaching session at the health unit closest to their geographical location. The same follow-up measurements (over 6 days) were conducted 6 weeks after the teaching intervention. A total of 25 intervention and control sessions occurred at roughly five-week intervals. After six-week data collection, the sleep group received the infant safety information and the safety group received the infant sleep information. We provided the safety group with infant sleep information because the literature indicates infants’ BSPs have negative outcomes for parents [10–12] and infants [4] and we regarded it as unethical to withhold a potentially useful intervention in the absence of any standard accessible treatment for infant BSP.

### Intervention

Our intervention aimed to improve a BSP at an age when infants are developmentally capable of sustaining a long self-regulated sleep period for, on average, 10 h [20]. We sought to change families’ cognitions and behaviors to promote infant self-soothing and targeted our intervention to both parents because both have affected infant sleep [21]. Parental cognitions are defined as beliefs, expectations, and interpretations of children’s sleep behavior which are viewed as influencing parent-infant interactions and infant sleep patterns [22, 23]. Because public health nurses frequently encounter families experiencing infant BSP we trained them to deliver the sleep intervention. Nurses also delivered the safety control sessions. The principal investigator created the materials for the teaching sessions and phone calls. Total treatment duration was 3 weeks: one 2-h teaching session followed by bi-weekly telephone calls for 2 weeks. The nurses who delivered the teaching sessions called the parent leading the intervention/control twice a week for 2 weeks to reinforce concepts and provide support. The intervention and control groups were asked the same questions during the telephone calls: How were parents managing; what strategies were they using; what were the effects of their strategies on their infants and the parents; and what, if any, difficulties were they experiencing?

#### Sleep intervention

Groups of families received information about normal infant sleep, effects of inadequate sleep for infants and parents, setting limits around infant sleep, the importance of daytime and sleep routines, and negative sleep associations (Table 1). The intervention introduced behaviors that families could use to promote infant self-soothing, in particular, controlled comforting, with two to 10 min taken to console the infant and then leaving the room for increments of two minutes up to 10 min intervals [24]. Families observed a recording of an infant in light and deep sleep (so they could see an infant vocalizing and opening her eyes while asleep) and an interview with parents living with a child with a sleep problem. The video was intended to point to longer term sleep problems when interventions were not initiated in infancy. Families also received handouts of the scripted power point presentation, weekly sleep-wake-feed-play charts (routines), and controlled comforting charts. Charts enabled them to track their use of techniques and any changes in their infants’ sleep.

**Table 1:** Topics addressed during teaching sessions

*Information about infants’ patterns (cognitions)*	
▪ Infants have developmental shifts	
▪ Infant feeding requirements at night	
▪ Sleep-wake-feed-play and usual sleep patterns and typical sleep progression	
▪ Infant behaviors associated with sleep types and tiredness cues	
*Negative Sleep Associations (cognitions)*	
▪ Movement to sleep	
▪ Feeding to sleep	
▪ Putting an infant to bed asleep	
▪ Reactive co-sleeping	
*Unrealistic expectations about sleep (cognitions)*	
▪ Less napping and late bedtimes will promote night sleep	
▪ Nap and bedtimes do not matter	
▪ Parents’ approaches can differ	
*Effects of sleep loss on infants and parents (cognitions)*	
▪ Associations between behavioral sleep problems and infant growth and development	
▪ Associations between sleep disturbance, parental mood, confidence, and problem-solving	
*Strategies to Reduce Night Waking (behaviors)*	
▪ Daytime, bedtime, and naptime routines	
▪ Minimal stimulation before bed	
▪ Feeding 20 min prior to settling	
▪ Controlled comforting with periodic checking	
▪ Avoid reactive co-sleeping	

#### Safety control

Groups of families learned about infant safety risks, e.g., shaken baby syndrome, choking, falls, sleep positioning, motor vehicle travel, pacifiers, suffocation, strangulation, poisoning, and burns, as well as factors influencing risks and strategies for prevention. Co-sleeping as a risk to infant safety was not discussed. Families received a handout of the scripted power point presentation, which included a safety checklist. Nurses answered questions that were raised during the teaching session, e.g., recalls for cribs or infant car seats.

### Training and intervention fidelity

Ten public health nurses, with five or more years of experience, and three registered nurses, with two or more years of experience in community settings, were separately trained during eight hour sessions for the sleep intervention (Table 2) and safety control. Role plays for delivering the teaching session and follow-up telephone calls were included. Nurses received training manuals, documents with frequently asked questions and answers about sleep and safety, standard scripted formats for the teaching sessions and follow-up calls, and a PowerPoint Module. Public health nurses received videotaped materials. Ad hoc calls to participants by nurses were prohibited.

**Table 2:** Training program for sleep intervention nurses

*Content*	
▪ Circadian rhythms & sleep cycle characteristics	
▪ Normal sleep amount & patterns for 6-to-12 month-olds	
▪ Developmental shifts in infant sleep and unrealistic parental expectations	
▪ Effects of sleep fragmentation & short cycles	
▪ Routines daytime & pre-sleep	
▪ Negative sleep associations	
▪ Effects of sleep problems on infants	
▪ Effects of sleep problems on parents	
▪ Trials and reviews of behavioral sleep interventions	
▪ Strategies to reduce night waking	
▪ Screening infants for conditions requiring medical referral	
*Skills*	
▪ Screening infants for conditions requiring medical referral	

Nurses video-recorded their teaching sessions and audio-recorded their follow-up calls for review. The principal investigator reviewed 100 % of the initial training sessions and follow-up calls to provide support and reinforce standardization. Thereafter, the principal investigator randomly reviewed 50 % of videos and 10 % of the follow-up call recordings. In 90 % of teaching sessions and 80 % of calls, nurses followed protocols. The principal investigator worked with nurses omitting prescribed behaviors to incorporate them. The information from the phone calls is not included in this report.

### Outcome measures

Our primary outcome was a composite measure of significant sleep disturbance consisting of either parent reporting a severe sleep problem or mean actigraphic wakes of ≥ 2 per night averaged over 5 nights. Evidence that the intervention was successful would be significantly smaller proportions of parents reporting severe infant sleep problems (four-point [no problem, mild, moderate, severe] measure) or significantly smaller proportions of infants with mean actigraphic wakes of ≥ two per night in the intervention group compared with controls. Infant sleep problems are not confined to night waking per se; they also include parents’ interpretations of infant night waking and signaling [23].

The severity measure was taken from the Longitudinal Study of Australian Children - a large nationally representative study using primary caregiver report on a scale about children’s sleep (no problem, or mild, moderate, or severe problem) [6, 11, 25, 26]. Australian parents’ reports of sleep problem severity were robust indicators of frequent night waking [26]. The Ambulatory Monitoring Micromini-motion logger® was used in a zero crossing mode [27], with upgraded Action-W® version 2.7 software to collect and analyze actigraphy data [28]. The actigraphs were worn on infants’ left ankles for 6 days. The software incorporates Sadeh’s algorithm [28] for differentiating sleep and wakes in infants less than one year of age. Most studies employing actigraphy reported using Ambulatory Monitoring actigraphs [29]. Actigraphic data have had high rates of agreement with polysomnography (PSG) recordings [30, 31]. Following downloading, a physician, blinded to group assignment, scored all of the actigraph data. Families also completed an infant sleep diary and a form with settling and rising and actigraph removal and reapplication times to assist with interpretation of the actigraphy data. Sleep diaries have been used in large longitudinal studies to determine children’s sleep duration and night waking [32].

Our secondary outcomes were infants’ longest sleep time in minutes (actigraphy) and mean wakes (actigraphy and diary) and parents’ experiences, using the Multidimensional Assessment of Fatigue Scale [MAF] [33], Center for Epidemiologic Studies Depression Measure [CESD] [34], Pittsburgh Sleep Quality Index [PSQI] [35], and Maternal (parental) Cognitions about Infant Sleep Questionnaire [MCISQ] [36], at six weeks post-teaching session. For psychological measures, higher scores indicate more difficulty. There is support for validity and reliability; instrument testing occurred in the pilot study [14, 15]. For the trial, Cronbach’s alphas were: MAF 0.94, 0.95; CESD 0.90, 0.89; PSQI 0.64, 0.53; MCISQ 0.79, 0.84. The MCISQ has not been validated with fathers; however, Morrell’s items were developed from therapy vignettes for couples experiencing an infant sleep problem [36] and other studies have used the measure with mothers and fathers [22, 23]. Our original secondary hypothesis specified significantly improved changes in experimental versus control group parents’ experiences; however, we capitalized on data from both parents by conducting a post hoc analysis comparing primary and secondary caregivers separately.

### Statistical analyses

Our sample size was based on detecting an arithmetic difference of 20 % in outcome proportion reporting severe sleep problems between the intervention and control groups [6], using results obtained by Hiscock and colleagues’ (2007) who reported 70 % in control group and 50 % in intervention group. That absolute difference required 97 subjects per group to obtain power of 0.80 in a two-sided test with type I error rate of 0.05. Allowing for loss of follow up of up to 20 %, our planned sample size was 240 families; we recruited and randomized a total of 235 families. We did not incorporate secondary outcomes in our power calculation. No interim analysis was planned.

The primary analyses were conducted on an intention-to-treat basis considering a two-tailed p-value ≤ 0.05 as significant and using R (version 2.13.0). We compared observed proportions of parents reporting severe sleep problems and actigraphic wakes of ≥2 per night between groups using a Fisher’s exact test, with adjustment for baseline sleep problem severity using the Mantel-Haenszel test, including corresponding 95 % confidence intervals for differences in proportions or relative risks. Means for continuous outcomes were compared using linear (both fixed and mixed effects) model analyses, with adjustment for baseline measurements. We adjusted our analysis of sleep problem severity for infant gender and feeding status.

Because families were assigned to groups associated with particular health centers and providers we examined health center and provider effects on primary outcomes using mixed effects logistic regression. In addition, we generated multiple imputations for incomplete data using iterative regression imputation [37]. The results reported are based on the available data. Multiple imputation was undertaken to confirm the validity of the available data analysis.

## Results

We screened 386 families for study eligibility (Fig. 1). Sixty-eight infants (17.6 %) were excluded because they were: outside the age range (*n* = 28), had chronic health problems (*n* = 2), or did not meet the BSP criteria (*n* = 38). Thirty-eight parents (9.8 %) were excluded because they lived outside the study catchment (*n* = 10), were unavailable for training sessions (*n* = 5), or had diagnoses of depression or sleep apnea (*n* = 23). Twenty-seven families (7 %) refused participation because they regarded data collection as too onerous. Of 253 families (66 %) that agreed to baseline data collection, 18 (7 %) could not be randomized due to infant illness (*n* = 1), family bereavement (*n* = 1), parents separating (*n* = 1), and regarding the study as too onerous (*n* = 15). Two hundred and thirty-five families (462 parents) were randomized to the intervention and control groups. At six weeks, 110 families (206 parents) were in the intervention group and 108 families (204 parents) were in the control group. Loss to follow-up at the six-week outcome collection point was 6 % in the intervention group and 7.4 % in the control group.

At baseline (Table 3), parents had a mean age of 35 years, a mean of 17 years of education, and most had a partner. Most families had one child. Family income ranged from $10,000 to ≥ $110,000 per annum, with about 40 % of the sample reporting incomes between $10,000 and $89,999 CAD per annum. The majority of the sample self-identified as Canadian, with the next largest groups reported as European, Chinese, and South Asian. The groups were similar except there were more male infants and fewer breastfeeding infants in the intervention group compared with the control group. Both parents attended the sleep session in 86 % of families and the safety session in 62 % of families. An average of nine parents per group attended each sleep and safety teaching session. For follow-up telephone calls, 98 % of sleep group and 85 % of safety group families received the first call, 96 % and 80 % received the second call, 94 % and 80 % received the third call, and 86 % and 79 % received the fourth call. The sleep group averaged 3.7 calls per family and the safety group 3.3 calls per family.

**Table 3:** Baseline demographic variables for infants and parents

Category	Sleep intervention	Safety control
Infants	*n* = 117	*n* = 118
Age, mean (SD), mo	6.7 (0.92)	6.8 (0.96)
Male, No. (%)	74 (64)	57 (48)**
Breastfed, No. (%)	99 (85)	113 (96)**
Parents	*n* = 229	*n* = 233
Age, mean (SD), y	35.5 (5.6)	35.4 (5.1)
Married or in stable relationship, No. (%)	110 (97)	110 (97)
Number of children, mean (SD)	1.3 (0.7)	1.3 (0.7)
Education, mean (SD), y	17 (2.8)	17 (2.8)
Family Income, Canadian dollars, No. (%)		
$10,000 - 29,999	12 (6)	8 (4)
$30,000 - 59,999	22 (11)	42 (20)
$60,000 - $89,999	36 (17)	42 (20)
$90,000 -109,000	56 (27)	32 (15)
≥ $110,000	81 (39)	91 (42)^a^
Cultural Identity, No. (%)^b^		
Canadian	111 (50)	123 (54)
Chinese	23 (10)	19 (8)
European	30 (14)	25 (11)
South Asian	21 (9)	20 (9)
Other	38 (17)	42 (18)

^a^Rounding error. ^b^Parents self-determined cultural identity

Note: **p < .01

### Primary outcome

Incomplete actigraphic data (missing epochs due to signal loss) for a 24-h period were excluded from analysis and occurred similarly across intervention and control groups (7.6 % and 8.1 % respectively). At six weeks post-intervention, 96 actigraphy records were analyzed for each group. Ninety-four control group infants had an average of ≥2 wakes per night compared to 93 intervention group infants, a risk difference of -0.2 % (95 % *CI*: −1.32, 0.91). At six weeks, 4 % of infants in the intervention group (*n* = 4) compared to 14 % of infants in the control group (*n* = 15) had parental reports of severe sleep problem scores (relative risk of 0.26 [95 % *CI*: 0.09, 0.77]); adjusting for baseline scores yielded an estimate of 0.30 (95 % *CI*: 0.11, 0.84) and a risk difference of -10% (95% *CI*: -16.8, -2.2) (Table 4). No estimates were substantially affected by adjustment for baseline characteristics. Adjusting for infant gender and feeding status did not alter the sleep problem severity results. There was no indication of pertinent variation between centers or providers.

**Table 4:** Infant sleep characteristics at baseline and 6 weeks

	Sleep intervention		Safety control		Adjusted difference at outcome	95 % confidence interval	*P*-value
	Baseline	6 Week	Baseline	6 Week			
Actigraphy	*n* = 113	*n* = 96	*n* = 109	*n* = 96			
Night wake episodes	8.2 (3.8)	7.9 (5.4)	8.8 (3.4)	7.7 (4.3)	−0.2	−1.32 to 0.91	0.72
Long wake episodes	4 (1.5)	3.2 (1.6)	4.2 (1.4)	3.2 (1.2)	0.02	−0.35 to 0.4	0.91
Longest sleep period	164 (46.2)	204.4 (87.5)	168 (53.2)	188.1 (50.2)	20.02	0.48 to 39.56	0.05
Sleep diary	*n* = 114	*n* = 106	*n* = 116	*n* = 106			
Night wake episodes	3.1 (1.2)	1.7 (1)	3.1 (1.2)	2.2 (1.1)	−0.45	−0.7 to −0.19	<.001
% infants with sleep problem (average = 2 wakes per night)	93 (81.6 %)	33 (31.1 %)	96 (82.8 %)	64 (60.4 %)	−29.9 %	−43.63 to −16.22	<.001
Parent report	*n* = 117	*n* = 108	*n* = 118	*n* = 107			
% of infants with severe night waking	32 (14 %)	4 (4 %)	44 (18 %)	15 (14 %)	−10 %	−16.8 to −2.2	0.01

Descriptive statistics are reported as Mean (SD) or n (%)

### Secondary outcomes

At six weeks, there was no difference between the intervention and control groups for mean change in actigraphic wakes or long wake episodes adjusted for baseline; however, there was a significant increase in the intervention compared to control infants’ longest sleep time (Table 4). Diary data were provided for 106 intervention and 106 control infants. Parents recorded significantly fewer wakes for intervention infants than control infants; 31.1 % of intervention infants compared to 60.4 % control infants had ≥2 night wakes (Table 4).

After adjusting for baseline, change in parents’ psychological measures indicated significant improvements for the intervention compared to the control group (Table 5). Intervention group parents’ fatigue, sleep quality, and depressed mood improved significantly compared to control group parents. As indicated, we conducted a post hoc analysis to compare changes in primary and secondary caregivers’ psychological variables between groups, after adjusting for baseline. For the intervention group, both primary and secondary caregivers’ sleep quality and fatigue improved significantly compared to the control group (Table 6). Depression improved for the primary caregivers in the intervention group compared to the control group but not for secondary caregivers.

**Table 5:** Comparison of intervention and control group parents for psychological variables

	Baseline mean (SD) (n)	6 week follow-up mean (SD) (n)	Baseline adjusted difference	95 % confidence interval	*P*-value
Fatigue MAF^a^					
Sleep intervention	26.8 (9.2) (*n* = 206)	18.7 (8.1) (*n* = 206)	−3.7	−5.74 to −1.68	0.001
Safety control	27.1 (8.6) (*n* = 204)	22.3 (9.2) (*n* = 204)			
Depression CES-D^b^					
Sleep intervention	13.8 (9) (*n* = 206)	9.4 (7.7) (*n* = 206)	−2	−3.7 to −0.4	0.02
Safety control	15.4 (10.2) (*n* = 205)	12 (9) (*n* = 205)			
Sleep quality PSQI^c^					
Sleep intervention	8.1 (3.6) (*n* = 206)	5.7 (3) (*n* = 206)	−0.88	−1.5 to −0.2	0.009
Safety control	8.3 (3.4) (*n* = 201)	6.5 (3.3) (*n* = 202)			
Cognitions MCISQ^d^					
Sleep intervention	*n* = 223	*n* = 205			
Safety control	*n* = 230	*n* = 205			
Sleep doubts^e^					
Sleep intervention	7.2 (4.1)	4.3 (3.6)	−1.3	−2.0 to −0.6	<.001
Safety control	6.9 (4.2)	5.5 (4)			
Sleep anger^f^					
Sleep intervention	6.8 (3.4)	5.2 (3.1)	−1.1	−1.8 to −0.4	0.003
Safety control	7.3 (4)	6.5 (3.8)			
Sleep and feeding^g^					
Sleep intervention	7.3 (3.6)	3.9 (3.4)	−1.4	−2.1 to −0.7	<.001
Safety control	6.9 (3.6)	5.2 (3.3)			
Setting sleep limits^h^					
Sleep intervention	14.9 (4.9)	10.1 (4.8)	−2.48	−3.5 to −1.5	<.001
Safety control	14.9 (4.9)	12.4 (5.2)			
Sleep safety^i^					
Sleep intervention	2.9 (2.5)	1.9 (2)	−0.3	0.7 to 0.1	0.12
Safety control	2.7 (2.4)	2.2 (2.3)			

^a^Multidimensional Assessment of Fatigue Scale (global score range 1–50; higher scores indicate greater fatigue)

^b^Centre for Epidemiologic Studies Depression Measure (score range 0–60, higher scores indicate worse depression)

^c^Pittsburgh Sleep Quality Index (global score range 0–21; higher scores indicate worse sleep quality)

^d^Maternal Cognitions about Infant Sleep Questionnaire (higher scores indicate more parent difficulty)

Subscale score range for: ^e^Doubts about managing sleep = 0–25, ^f^Anger about infant sleep = 0–25

^g^Managing infant sleep and feeding = 0–15, ^h^Setting i nfant sleep limits = 0–25, ^i^Infant sleep safety = 0–10. The means were adjusted for baseline

**Table 6:** Comparison of intervention and control group by caregiver on psychological variables at 6 weeks

Variable & caregivers	Sleep intervention		Safety control		Baseline adjusted difference	95 % confidence interval	*P* value
	Mean (SD)	n	Mean (SD)	n			
Fatigue MAF^a^							
Primary caregiver	19.3 (8.1)	108	23.5 (9.2)	105	−4.2	−6.45 to −1.96	<.001
Secondary caregiver	18.1 (8.1)	98	21.1 (9)	99	−2.7	−4.90 to −0.50	.02
Depression CES-D^b^							
Primary caregiver	9.1 (7.9)	108	12.5 (8.4)	106	−2.87	−4.81 to −0.93	.004
Secondary caregiver	9.7 (7.5)	98	11.5 (9.7)	100	−0.77	−2.89. to 1.36	.48
Sleep quality PSQI^c^							
Primary caregiver	6.2 (3.1)	108	7.1 (3.4)	107	−0.88	−1.70 to −0.06	.04
Secondary caregiver	5.1 (2.8)	98	5.9 (3.1)	95	−0.72	−1.45 to 0.01	.05
Cognitions MCISQ^d^							
Sleep doubts^e^							
Primary caregiver	4.6 (3.5)	108	5.9 (4)	107	−1.56	−2.43 to −0.68	<.001
Secondary caregiver	3.9 (3.7)	97	5.1 (4.1)	98	−1.26	−2.14 to −0.39	.005
Sleep anger^f^							
Primary caregiver	5 (3.3)	108	6.6 (4.2)	107	−1.37	2.25 to - 0.50	.002
Secondary caregiver	5.3 (2.9)	97	6.3 (3.3)	98	−0.75	1.54 to 0.31	.06
Sleep and feeding^g^							
Primary caregiver	4.5 (3.6)	108	6 (3.3)	107	−1.59	2.41 to - 0.77	<.001
Secondary caregiver	3.2 (3.2)	97	4.4 (3.2)	98	−1.4	2.19 to - 0.61	<.001
Setting sleep limits^h^							
Primary caregiver	10.8 (4.8)	108	13.5 (5.2)	107	−2.63	−3.80 to −1.47	<.001
Secondary caregiver	9.2 (4.8)	97	11.1 (4.9)	98	−2	−3.12 to −0.88	<.001
Sleep safety^i^							
Primary caregiver	2.2 (2)	108	2.5 (2.2)	107	−0.42	0.91 to 0.07	.09
Secondary caregiver	1.7 (2)	97	2 (2.3)	98	−0.3	0.80 to 0.20	.25

^a^Multidimensional Assessment of Fatigue Scale (global score range 1–50; higher scores indicate greater fatigue)

^b^Centre for Epidemiologic Studies Depression Measure (score range 0–60, higher scores indicate worse depression)

^c^Pittsburgh Sleep Quality Index (global score range 0–21; higher scores indicate worse sleep quality)

^d^Maternal Cognitions about Infant Sleep Questionnaire (higher scores indicate more parent difficulty)

Subscale score range for: ^e^Doubts about managing sleep = 0–25, ^f^Anger about infant sleep = 0–25

^g^Managing infant sleep and feeding = 0–15, ^h^Setting infant sleep limits = 0–25, ^i^Infant sleep safety = 0–10. The means were adjusted for baseline

When we examined parental cognitions about sleep, after controlling for baseline, there was a significant improvement in all areas of cognition, except for sleep safety, on comparing intervention parents with controls (Table 5). In the post hoc analysis we analyzed data for primary and secondary caregivers separately. For both primary and secondary caregivers the intervention groups’ doubts about managing infant sleep, comfort managing sleep and feeding, and comfort setting limits around infant sleep improved significantly compared to the control group (Table 6). Primary caregivers’ cognitions of anger about infant sleep but not secondary caregivers’ cognitions improved significantly for the intervention group compared to the control group. There were no significant differences in intervention group primary or secondary care providers’ concerns about sleep safety compared to the control group.

## Discussion

Given the frequency of BSPs in infancy and their negative long-term effects on parents and children [1–9] brief and effective interventions to manage BSPs are important. Ninety percent of American school-aged children have received less than the recommended hours of sleep [38]. Finding ways to reach parents and assist them to recognize BSP as amenable to change early in children’s development can potentially improve parents’ knowledge about sleep, factors influencing sleep, and approaches to managing children’s sleep. This is the first trial of the efficacy of a cognitive-behavioral group intervention (CBGI) for parents with infants from 6- to 8-months of age with BSPs. While the control group also improved over time, our principal findings (adjusted for baseline) indicated a significant improvement in parents’ perceptions of the severity of the infant sleep problem, reduction in numbers of night wakes by sleep diary, increase in length of longest night sleep by actigraphy, and improvement in parents’ cognitions about infant sleep, fatigue, sleep quality, and depression in the intervention group compared to the control group.

The results, including no significant reduction in night waking by actigraphy, support the efficacy of our intervention and our goal to assist parents to accept normal infant arousal behavior by understanding the effects of self-soothing. The diary data demonstrated that a clinically significant proportion of infants in the intervention group managed to return to sleep following typical night arousals without signaling their parents. Behavioral interventions are intended to assist infants to self-soothe back to sleep rather than to prevent night waking. In the intervention content we emphasized links between parental responses to infants’ crying and signaling and infants’ failure to learn to self-soothe to return to sleep following typical night waking. Parental responses to infant signaling can interfere with self-soothing and extend the duration of infants’ night waking [39].

Adjusting for baseline, there was not a significant improvement in actigraphy night waking in the intervention group compared with the controls, even when we compared the groups on long wakes. Our results fit with emerging research suggesting that actigraphs lack specificity to detect wakes in infants [29]. Actigraphy data have shown low specificity for children’s wakes but high intraclass correlations with polysomnography for sleep duration [40].

The differences between primary and secondary caregivers’ improvement in depression and sleep anger fit with findings of other studies. For example, Hiscock and Wake reported that mothers who regarded their infants’ sleep as a problem were more likely to have depression and depression scores increased with sleep problem severity [41]. Another study reported significant correlations between maternal sleep cognitions and depression scores, with mothers of children with sleep problems scoring significantly higher on anger about sleep than mothers of control children [42]. In our study, primary caregivers were the parents engaging with settling infants to sleep, night waking, and the intervention.

### Strengths and limitations

The trial responded to a number of criticisms of sleep interventions [13] by offering a small group format, a relatively brief intervention, a combination of objective and subjective sleep measures, and a standardized definition of a behavioral sleep problem [19]. The trial was preceded by a pilot study [14, 15] and incorporated a standardized intervention through formal training of nurses, scripted interventions, systematic review of intervention fidelity, and less than 10 % attrition at primary outcome. In the pilot study [14, 15], the principal investigator delivered all of the sleep interventions for the seven groups of parents and provided all of the telephone support thereby ensuring fidelity was maintained. We created similar exposure to attention for the sleep and safety groups and blinded our participants to our hypotheses [43]. We incorporated secondary care providers (fathers) in our trial in contrast to previous foci on mothers alone [21], given evidence both parents influence children’s sleep patterns [22].

Exclusion of parents who did not read or speak English and research participant characteristics (older, well-educated and high income) limits the generalizability of results. It is interesting that our sample is relatively representative of the population being served. About 40 % of our sample reported average family incomes of between $10,000 and $89,999 CAD; these family income levels are relatively common in the city where the study was conducted. In 2011, the mean and median household incomes for Metro Vancouver were $83,666 CAD and $63,347 respectively [44] and median couple income in Vancouver in 2012 was reported as $76,690 CAD [45]. In Vancouver in 2011, 60 % of adults reported university education or college diplomas [46].

Lack of blinding for parent participants had the potential to bias the study results. We asked about other resources parents accessed but did not place limits on parents’ access to other forms of help, such as sleep consultants. We did not control for parents’ access to other services because we intended the trial to resemble public health nurses’ practice where parents would have unlimited access to other resources. The lengthy recruitment period increased the possibility that control group parents were contaminated by conversing with parents, who received the trial intervention. Both lack of parental blinding and contamination of the control group could potentially have contributed to underestimating the effect size of the outcome. Some parents might prefer one-to-one rather than group formats to assist with problems.

We did not compare intervention and control groups for sleep-onset difficulties in our study for several reasons. At baseline, parents were often rocking or feeding their infants to sleep. Thus, the actigraphic mean for sleep onset latency at baseline was 0.58 min. Parents often reported settling their infants after they specified sleep onset on written diaries. In other words, infants were asleep when parents placed them in their cribs. Infants are unable to indicate when they try to initiate sleep so we suggest that the standard definition of sleep latency does not apply to this developmental group.

We were not surprised that, despite significant differences between groups on infant gender and breastfeeding status at baseline (Table 3), adjusting for their status did not affect our results for sleep problem severity. In large multinational studies, neither gender nor breastfeeding status has influenced night waking [47, 48]. It has been breastfeeding to sleep that has been associated with sleep problems [48] and our intervention recommended that parents refrain from feeding infants to sleep.

The availability of public health nurses trained in behavioral sleep interventions creates potential for nurses to offer interventions through contact with families attending group postnatal drop-ins. Not only can situating a short-term group intervention in public health units overcome barriers to families receiving help for common infant BSPs [49, 50] but also being exposed to other families experiencing an infant BSP decreases families’ sense of isolation and helps them regard their infants’ problems as common and amenable to change [16]. Contacting families by telephone for follow-up could be factored into public health nurses’ daily workload. Sleep consultants in the area routinely charge families about $250 to $450 per consultation, thus potentially limiting families’ access to such services. Parents’ changes in cognitions, observed in our study, suggest that their thinking changed when provided with evidence-based information about infant sleep and strategies to reduce sleep problems by skilled practitioners. Future studies could incorporate video surveillance of infant sleep, a more valid way of identifying insomnias [51], indicators of self-soothing, and attachment measures to provide evidence about effects of interventions on infants.

## Conclusions

Compared to parents in a control group, parents exposed to a CBGI experienced significantly improved perceptions of infant sleep, sleep cognitions, mood, sleep quality, and fatigue, but not number of wakes measured using actigraphy.

## Abbreviations

BSP: Behavioral sleep problem
CBGI: Cognitive-behavioral group intervention
CESD: Center for Epidemiologic Studies Depression Measure
MAF: Multidimensional Assessment of Fatigue Scale
MCISQ: Maternal (parental) Cognitions about Infant Sleep Questionnaire
PSQI: Pittsburgh Sleep Quality Index
